# The Young Women’s Health CoOp in Cape Town, South Africa: Study protocol for a cluster-randomised trial for adolescent women at risk for HIV

**DOI:** 10.1186/s12889-018-5665-5

**Published:** 2018-07-11

**Authors:** Wendee M. Wechsberg, Felicia A. Browne, Tara Carney, Bronwyn Myers, Alexandra Minnis, Robert MacDonald, Jacqueline W. Ndirangu, Leslie B. Turner, Brittni N. Howard, Nathaniel Rodman

**Affiliations:** 1 Substance Use, Gender, and Applied Research Program, RTI International, 3040 E. Cornwallis Road, Research Triangle Park, NC 27709-2194 USA; 2 Health Policy and Management, Gillings School of Global Public Health, University of North Carolina, Chapel Hill, NC USA; 3 Psychiatry and Behavioral Medicine, Duke University School of Medicine, Durham, NC USA; 4 Psychology in the Public Interest, North Carolina State University, Raleigh, NC USA; 5 Alcohol, Tobacco and Other Drug Research Unit, South African Medical Research Council, Tygerberg, South Africa; 6 Department of Psychiatry and Mental Health, University of Cape Town, Cape Town, South Africa; 7 Women’s Global Health Imperative, RTI International, San Francisco, CA USA; 8 School of Public Health, University of California, Berkeley, Berkeley, CA USA; 9 Western Cape Social Development, Western Cape Government, Cape Town, South Africa; 10 Research Computing Division, RTI International, Research Triangle Park, NC USA

**Keywords:** Adolescent women, Alcohol and other drug use, HIV prevention, Gender-based violence, School dropouts, South Africa

## Abstract

**Background:**

South Africa remains the global epicentre of HIV infection, and adolescent women have the highest incidence of HIV in the country. South Africa also has high rates of alcohol and other drug (AOD) use, violence, and gender inequality. Violence converges with AOD use, gender inequities and other disparities, such as poverty, to increase sexual risk and poor educational attainment for adolescent women. This study seeks to test the efficacy of peer recruitment and cofacilitation of the Young Women’s Health CoOp (YWHC), a comprehensive gender-focused intervention to reduce HIV risk behaviours and increase the uptake of HIV counselling and testing (HCT) among out-of-school, adolescent women who use AODs. The YWHC is facilitated by local research staff and supported by peers.

**Methods:**

This cluster-randomised trial is enrolling participants into two arms: a control arm that receives standard HCT, and an intervention arm that receives the YWHC. Participants are enrolled from 24 economically disadvantaged communities in Cape Town, South Africa. These geographically distinct communities serve as clusters that are the units of randomisation. This study uses adolescent peer role models and research field staff to recruit marginalised adolescent women. At baseline, participants complete a questionnaire and biological testing for HIV, recent AOD use, and pregnancy. The core intervention is delivered in the month following enrollment, with linkages to health services and educational programmes available to participants throughout the follow-up period. Follow-up interviews and biological testing are conducted at 6 and 12 months post enrollment.

**Discussion:**

The study findings will increase knowledge of the efficacy of a comprehensive HCT, gender-focused programme in reducing AOD use, victimisation, and sexual risk behaviour and increase uptake services for out-of-school, adolescent women who use AODs. The trial results could lead to wider implementation of the YWHC for vulnerable adolescent women, a key population often neglected in health services.

**Trial registration:**

Trial registration no: NCT02974998, November 29, 2016.

## Background

South Africa is the global epicentre of HIV infection, with an estimated 7.1 million people living with HIV [1]. HIV prevalence among South African youth is among the world’s highest [2], with prevalence rising rapidly throughout adolescence [3]. Adolescent women in South Africa are disproportionately affected by HIV, having the highest incidence in the country [4]. Young women aged 15 to 19 are estimated to have an HIV prevalence eight times that of young men in the same age group [5].

A major driver of HIV risk in this group is alcohol and other drug (AOD) use. AOD use among South African youth is a pressing public health concern [6], particularly the use of alcohol, cannabis, and methamphetamine (“tik”) among Cape Town youth [7–9]. The South African National Youth Risk Behaviour Survey (YRBS) found that among female high school learners in the Western Cape, 21.1% had used cannabis in their lifetime, with 12.1% reporting use in the past month; 6.6% reporting lifetime tik use; and 40.1% reporting having engaged in binge drinking in the past month [6]. These high rates of AOD use are particularly alarming because AOD use is a known contributor to the risks associated with acquiring HIV [7, 10]. Additionally, previous studies suggest that adolescents who use AODs are at an increased risk of dropping out of school [7, 11–14].

South Africa also has high rates of violence, including gangsterism and gender-based violence (GBV) [15–17]. Exposure to violence, particularly sexual violence, converges with AOD use, social gender inequities and other disparities, such as poverty, to increase sexual risk and poor educational attainment for adolescent women [16, 18–20]. These factors contribute to the high rate of school dropout among youth [14], with an estimated 44% of South African adolescents not completing high school [21, 22]. Cape Town, in particular, has a school dropout rate of 54.9% among adolescents [19].

Gender inequality plays a key role in school dropout among adolescent women [23, 24], who are often discouraged from taking advanced-level courses and are expected to maintain domestic roles [24]. Additionally, they often report experiencing GBV, sexual abuse, and teenage pregnancy; the inability to afford school fees or transportation; participation in gangs or being in relationships with gang members; and AOD use as reasons for dropping out of school [9, 16, 18, 23]. Having peers who use AODs and engage in risky sexual behaviours can also have a detrimental impact on school dropout [14].

Adolescent women who drop out of school contend with unique consequences. For example, they face a higher probability of heavy alcohol or illicit drug use, which exacerbates their risk for victimisation, pregnancy, and HIV [9, 14, 16, 25]. They may also face limited employment opportunities because of their lack of education or skills training [14]. Because of the need for financial stability, young women may become financially dependent on their partners or choose to support themselves, their families, or their AOD use through trading sex, sex with older men (often referred to as “blessers”), or involvement with gangs or having relationships with gang members [14, 26, 27].

Adolescent women in South Africa who drop out of school face multiple risks, including those fuelled by historical gender biases. For example, they may experience stigma in seeking services, as many clinics do not practise anonymity or they may treat women who use AODs with disrespect [28]. Additionally, this hard-to-reach, key population remains underserved by HIV and substance abuse rehabilitation programmes and health services [29].

To date, there have been no comprehensive, evidence-based HIV counselling and testing (HCT) programmes that address these intersecting contextual and behavioural risks for HIV among out-of-school, adolescent women who use AODs [30]. All of these barriers contribute to low HIV testing uptake and adherence among South African adolescents [31], with between 25% and 35% of adolescent women currently being tested for HIV [32, 33].

This study builds on a formative study (supplement to R01DA032061) that seeks to alleviate these barriers by using a combination of peer recruitment and outreach to increase uptake of HCT, and to test the efficacy of an adapted comprehensive gender-focused intervention, the Young Women’s Health CoOp (YWHC), which targets vulnerable adolescent women in disadvantaged communities in Cape Town who use AODs and have dropped out of school. The YWHC is based on an adaptation of several iterations of the original best-evidence Women’s CoOp intervention [34, 35]. The adapted South African iteration, the Women’s Health CoOp (WHC) is the only intervention that addresses the intersection between AOD use, victimisation, and sexual risks for HIV [36–40]. The WHC has been shown to be particularly effective in reducing AOD use and enhancing empowerment through HIV risk reduction and skills building.

## Methods/Design

### Objectives

One aim of this study is to train and evaluate adolescent peer role models contracted from the government to market and conduct community outreach with established outreach workers to recruit 500 out-of-school adolescent women who use AODs, and to cofacilitate the YWHC. Another aim is to test the efficacy of the YWHC for reducing AOD use, victimisation, and sexual risk behaviour (primary outcomes) and improving access to effective treatment and support services through linkages to care (substance abuse rehabilitation, HIV, sexually transmitted infections [STIs], antenatal and reproductive health services as secondary outcomes) relative to standard HCT for adolescent women.

### Study design

**Fig. 1:**
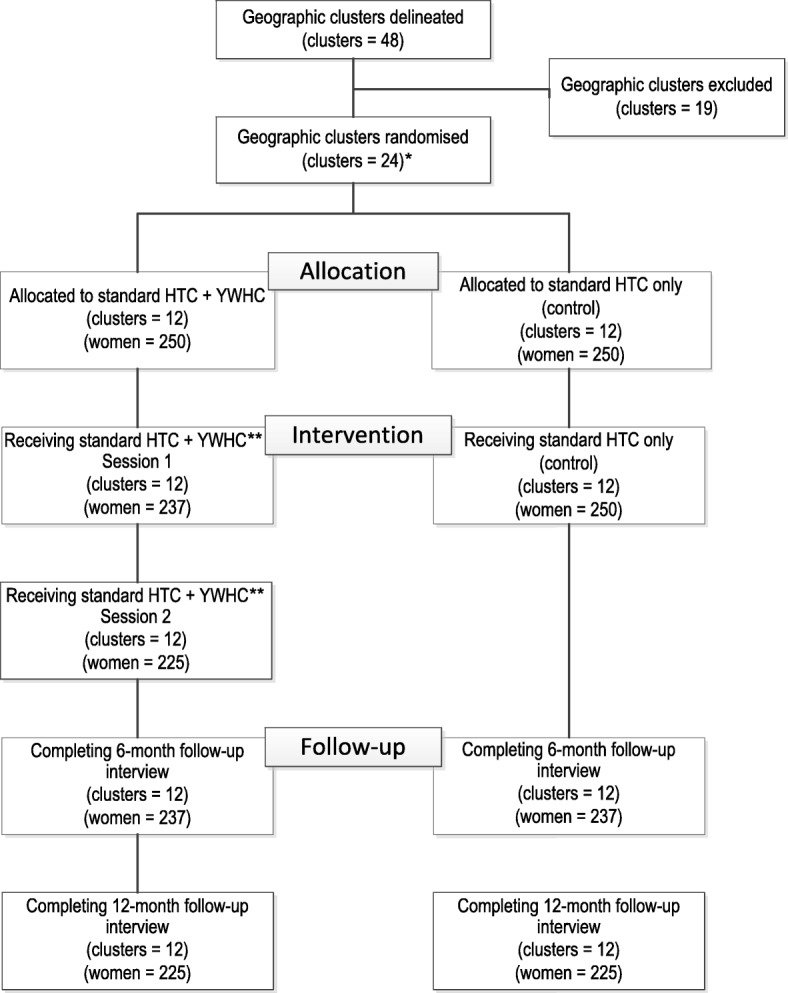
Study Design, with Estimated Recruitment.*An additional 5 clusters are available as replacement communities.**These group sessions occur approximately a week later, which accounts for some attrition.

This study is a cluster-randomised, two-arm field experiment conducted in 24 communities in Cape Town (Fig. 1). This cluster design was selected to reduce the likelihood of contamination between study arms.

### Cluster creation and selection

Two geographic areas were selected for the study, one predominantly Coloured (of mixed race ancestry) and the other predominantly Black African. These areas comprise numerous distinct communities that characterise race, population size, area size, and socioeconomic status. Consequently, each community constituted a potential cluster, with large communities subdivided into multiple clusters that yielded 48 clusters across the two geographic areas.

Clusters were selected for inclusion in the study using the following criteria:

1. There must be natural buffers between clusters; for example, at least two streets apart.
2. The predominant race/ethnicity in the cluster must be 90% of the residents or higher.
3. Very small clusters that could not generate the target sample size per cluster were excluded; for example, natural clusters with four or fewer small streets.

Clusters deemed outliers from other clusters were excluded; for example, high socioeconomic status in comparison to similar communities.

### Cluster randomisation

We selected 12 clusters for each of the two geographic areas. Within each block of 12, clusters were randomised to one of the two arms, with equal allocation. Additionally, 5 replacement clusters were selected (3 within the Coloured geographic area and 2 within the Black African geographic area). These replacement clusters will be used if any of the selected clusters are deemed unusable prior to conducting outreach and recruitment in a specific cluster. At times, gangs are very violent, making outreach and recruitment dangerous, even during the day. If this persists in a community, it will need to be replaced for the purposes of this study.

### Study population

Participant eligibility criteria include the following: (1) between 16 and 19 years old; (2) self-identify as a woman; (3) report consuming 2 to 3 alcoholic drinks at least once in the past 30 days *or* using illicit drugs at least once a week in the past 30 days; (4) be currently dropped out of school; (5) have been out of school for at least 6 months; (6) have not completed matriculation (high school) or do not have an N3 (National Technical Certificate) certificate; (7) report condomless sex with a male partner in the past 90 days; (8) report living in one of the 24 target disadvantaged communities; (9) have lived in the target community for at least 6 months; (10) plan to reside in the target community for the next year and can provide verifiable locator information; and (11) provide verbal and written consent. A short cognitive test is given at the end of the eligibility screener [41], and participants are excluded if they do not meet the minimum standard.

### Ethical approval

This study has been approved by the RTI International Committee for Protection of Human Subjects in the United States and the South African Medical Research Council’s (SAMRC) Ethics Committee in South Africa. All participants are required to provide informed consent prior to participating in the study. In the case of 16- to 17-year-old participants, assent is required, in addition to permission for consent by an older female community member who serves in loco parentis (in place of the parent). This approach has been used successfully in our previous South African study with adolescents (Carney et al.: Adolescent female school dropouts who use drugs and engage in risky sex: a brief pilot trial in South Africa, under review). Because of the sensitive nature of this study, in loco parentis enables the participant to select a female adult (either identified by the participant themselves or by the project staff), other than a parent, to provide consent on her behalf. This practise is used because if parents learn about a participant’s AOD use and sexual activity, it could result in conflict or violence in the family.

Once an older woman who will provide consent in loco parentis is identified, project staff schedule an appointment for the potential participant and the adult woman to conduct the consent process and provide written consent acting in loco parentis. Both the potential participant and her designated adult sit together during the initial consenting process. Following that, the potential participant is screened separately to confirm eligibility again, and she is assented separately from the adult woman to prevent coercion and to maintain confidentiality about sexual risk and AOD use. Any adult woman acting in loco parentis is also required to sign a confidentiality agreement form as part of the consent process so they understand that their experience at the project site is also confidential and that they must maintain confidentiality.

### Data and safety monitoring plan and data and safety monitoring board

This study utilises a Data and Safety Monitoring Plan (DSMP) to ensure the utmost protection of participants and their privacy. The DSMP includes a day-to-day procedural manual comprising data management and monitoring guidelines for project staff. Study data are kept confidential in a secure location, and identifiable information is stored separately from information with the participant’s alphanumeric study identification code (participant ID). All project staff are required to sign a confidentiality agreement, and they receive training on ethical roles and responsibilities in maintaining the privacy of participants. Procedures are in place to handle adverse events (AEs) or serious adverse events (SAEs), such as improper disclosure of information or mental or emotional discomfort. The study also utilises a Data and Safety Monitoring Board (DSMB) comprising three members: an expert in adolescent health, an infectious disease epidemiologist, and a bioethicist clinician. The DSMB meets every 6 months to review study progress and ensure adherence to the DSMP.

### Study procedures

#### Recruitment

The study uses female adolescent peer role models (aged 18 to 25) called Expanded Public Works Programme (EPWP) beneficiaries in combination with trained outreach workers to assist in reaching and recruiting participants. The peer EPWP beneficiaries are selected each year with help from the Western Cape Department of Social Development. They are trained on a series of topics, including professionalism, job expectations, AOD use, gender awareness, and community engagement. The EPWP beneficiaries also receive certification in a simplified Collaborative Institutional Training Initiative (CITI) training for human subjects and obtain training on completing daily outreach reports. Trained study outreach workers—who have worked in and around the target communities and are familiar with community outreach methods—offer oversight to the EPWP beneficiaries. The purpose of this team effort is to reach adolescent women who have dropped out of school and are using AODs. The trained study outreach workers conduct the screening with adolescents who they have identified during outreach, or the EPWP beneficiaries have referred them to, to ensure privacy and confidentiality. Study staff are multilingual in English and Afrikaans and/or isiXhosa.

Outreach is conducted in areas of high target population density in the selected communities. Trained EPWP beneficiaries and study staff post recruitment cards and fliers in their targeted communities, paying special attention to establishments where adolescent women who are out of school are known to frequent, such as community centres or libraries. Marketing about the study is conducted by walking through the neighbourhoods looking for adolescent women who may be hanging out near tuck shops (small, food-selling vendors), corners, and hair and nail salons; and by talking with community members. Potential participants who either contact project staff using the numbers given on the recruitment cards and fliers or who are approached through outreach are screened for eligibility by study staff.

#### Intake assessment

Participants are transported to a secure field site for all study activities. After final eligibility is determined through a rescreening, consent and/or assent is obtained, participants take a breathalyser test for recent alcohol use, locator information is collected, and a photograph of the participant is taken (which is returned at the final appointment). At this first appointment, participants complete the baseline questionnaire via computer-assisted personal-interviewing (CAPI) technology, and audio computer-assisted self-interviewing (ACASI) in their home language (English, Afrikaans, or isiXhosa) for sensitive topics, such as experiences of abuse. Staff then collect biological specimens to conduct pregnancy testing, screening for recent drug use, and rapid HIV testing. A urine specimen sample is collected to perform rapid tests to detect metabolites of recent use of cocaine, methamphetamines, amphetamines, opioids, ecstasy, and marijuana via a 5-panel drug screening test. Methaqualone (Mandrax) is measured through a separate urinalysis test. Two rapid tests are conducted for HIV testing; if a participant has a positive test result for either test, she receives a third test and posttest counselling in accordance with the national South African standards. If two tests show a positive HIV result, the participant is considered to have a positive test. All staff undergo training for biological specimen collection and are certified to conduct rapid HCT. All positive HIV screening determinations or positive test results for pregnancy are referred to the local health clinic regardless of study arm. For other services needed, participants in the intervention arm receive active referrals, whereas participants in the HCT control arm receive passive referrals.

#### Modifications to the protocol

We modified the protocol to improve the study design and to implement follow-up study procedures. Table 1 summarises these amendments, all of which were approved by the appropriate ethical boards. Each modification is explained in the table.

**Table 1:** Modifications to the Study Protocol

Date of Approval	Modification
August 2016	Reduced incentive amounts. Participants who have two positive (reactive) test results for their screening tests will not undergo a third (confirmatory) test. Increased the number of clusters from 12 to 24. Revised the screener, consent forms, and questionnaire to reflect protocol changes and the supplemental forms to reflect feedback during instrumentation programming and testing.
October 2016	Young women who cannot sign their signature or do not have a signature, will be asked to provide an inked fingerprint in place of a signature in accordance with South African Good Clinical Practice (GCP) guidelines. The alcohol breathalyser test was moved to the beginning of the intake appointment to have a valid assessment of alcohol use. Case management will consist of calls or in-person check-ins. Revised the consent form and locator form to reflect changes regarding case management and the baseline questionnaire and significant individual contact form based on instrument programming and testing.
April 2017	Addition of 6-month follow-up protocol: Submission of the 6-month follow-up consent form and 6- and-12-month questionnaire.
October 2017	Addition of 12-month follow-up protocol: Submission of the 12-month follow-up consent form. Updated all previously approved consent forms and the Rights of Research Participants Card to reflect change in chairperson.
November 2017	Made a minor change to the 12-month follow-up consent form. Edited the ACASI section of the baseline and follow-up instruments, which included adding referral prompts related to self-reported violence experienced in the past 3 months.

### Study activities by arm

#### Intervention arm

Participants in the intervention arm take part in all of the activities described above for the control arm. Additionally, they are asked to attend two group workshops of the YWHC. Participants are contacted by staff to confirm attendance to the intervention workshops within a week of their intake appointment. Up to eight participants form a workshop group.

The intervention consists of two group workshops lasting approximately 2 h each, with each workshop containing several modules and exercises (see Table 2 for a detailed summary of the workshop content). Workshops are conducted approximately 7 to 10 days apart. Workshop 1 covers developmental issues, AODs, pregnancy and parenting, gender issues, violence, communication, and negotiation. Workshop 2 covers sex and risk, HIV and other STIs, sexual risk reduction (including male and female condoms), and safer sex negotiation with partners. The modules include voices and quotes from the formative focus group discussion participants who willingly shared their stories. Both workshops end with each participant working toward developing a personalised risk-reduction action plan. As with the intake, active referrals are offered where appropriate.

**Table 2:** Young Women’s Health CoOp Workshop Content

Workshop 1		
Topic	Slides	Activity
Becoming a Woman	• Life Stages of Development • Who Influences You • Growing up and Maturing • How Can You Be in Control? • Becoming Your Own Woman • Making Decisions and Never Too Late to Change	
Alcohol and Other Drug (AOD) Use	• AOD Use Compromises Young Women • Intersecting Risks • Using Alcohol and Other Drugs • Types of AODs and Risks • Effect of AOD Use on Sex • Risks of Using AODs • Reducing AOD Risks	Drug Type Activity
Pregnancy and Parenting	• Breastfeeding • Teen Parenting Challenges • Tips for Being a Good Parent	
Communication and Negotiation	• Learning How to Communicate and Negotiate With Others • Understanding Conflict • Conflict Resolution Steps • Ground Rules for Conflict Resolution	Conflict Resolution Role-play
Gender and Power	• Inequality and Lack of Power • Types of Boyfriends • Getting Away from Bad Boyfriends • Gangs and Issues • When Things Go Wrong • Response to Danger with Guys	
Violence	• Intersecting Risks • Rape and Sexual Assault • Truths and Myths of Rape • Safety Tips When Going Out	Truths and Myths of Rape
Behaviour Change	• Resources for Change • Identifying Support and Healthy Role Models • Dreams and Goals	
Personalised Action Plan	• Reduce AOD use • Conflict Resolution and Violence Prevention • Improve Life Situation	Complete Workshop 1 Goals and Steps in Booklet
Workshop 2		
Topic	Slides	Activity
HIV	• Why Reaching Young Women Is Important • What You Need to Know • How Do Young Women Get Infected with HIV? • What Is HIV? • What Young Women Say about HIV Testing • Window Period • If Someone Is Infected with HIV • Breastfeeding and HIV	
Sex and Risk	• Intersecting Risks • Levels of Sexual Risk • Relationships and Expectations • Sexual Pressure • Unprotected Sex	Sexual Risk Activity
Male and Female Anatomy	• Examining Body Parts • Keeping Private Parts Healthy • Male Anatomy	
STIs	• STI Facts • Types of STIs • How to Get Help • Preventing HIV and Other STIs	Treatable versus Curable STIs
Male and Female Condoms and Protection	• Male Condom and How to Use It • Female Condom and How to Use It	Male and Female Condom Demonstration and Practise
Other Sexual Activities and Risks	• Oral Sex and Clingwrap/Oral Dams • Exchanging Sex • Reducing Sex Risks • How to Stay Healthy	
Safer Sex Negotiation	• Being Strong, Safe, and Healthy Young Women • Tips for Effective Refusal for Not Having Sex • How to Talk With “The Guy” About Safer Sex • Negotiating with Different Partner Types	
Personalised Action Plan	• Protect Health • Improve Life Situation	Complete Workshop 2 Goals and Steps in Booklet

#### Control arm

Participants in the control arm receive the standard HCT protocol performed in South Africa, as mentioned in the intake assessment. Additionally, participants obtain referrals upon request to other health services, except in the case of pregnancy or HIV-positive screening determination.

### Participant satisfaction forms

When participants complete the intervention, they are asked to reflect on their experience by completing a Participant Satisfaction Form by themselves. This information provides feedback on how effective the YWHC was for each participant who completed both workshops. The form asks questions about what the participant learned, the changes they made, and for suggestions regarding the aspects of the intervention they would change or improve. The form also asks about the usefulness of the personalised action plan in helping participants improve their life and if they would encourage others to attend the workshops.

### Case management of personalised action plan goals

After completion of the second workshop, staff follow up with participants individually to assist them with attaining the goals developed in their personalised action plan. Staff check in approximately monthly via mobile phone or in person. Because a majority of participants do not have mobile phones, most of the case management is conducted in person.

### Fidelity of the intervention

The fidelity of the intervention workshops is assessed by audio-recording the workshops, with the consent/assent of all participants. The South African Project Director or designee listens to all of the recordings and provides feedback to the interventionists in the following areas: (1) coverage of all the activities that should be included in the two workshops, which is an assessment of whether the interventionist covers all the relevant topics, conducts all the activities, and ensures that the skills are practised by participants; and (2) delivery of the workshop to ensure that the interventionist covers the material on the curriculum, maintains focus during the group workshop, and monitors time to ensure that the workshop is not too lengthy. The assessment also includes the style in which the interventionist provides the workshop; for example, if she was nonjudgmental in her approach, asked participants questions, and could answer the questions. The South African Project Director also checks whether the personalised action plans are completed by each participant that attends the workshops, and whether the goals are concrete and measurable.

### Follow-up assessment

Participants in both study arms return for 6- and 12-month follow-up assessment, which includes reconsenting, updating locator information, administering a follow-up questionnaire, and biological testing. Each of these two appointments lasts approximately 1.5 h. If a participant tests positive for HIV at baseline, they are not tested again for HIV at follow-up.

### Outcome measures

The primary outcomes of this study are reduced AOD use (as measured by AOD testing and by self-reported frequency of AOD use) and reduced sexual risk (as measured by condomless sex, self-reported number of casual partners, sex trading, and partner concurrency) in the past 30 days. We used the Revised Risk Behavior Assessment (RRBA) with youth measures to assess these outcomes. An additional outcome is reduced violence and victimisation in the last 3 months [42].

The secondary outcomes of this study include access to health services, as measured by self-reported use of sexual reproductive health services and HIV and STI testing services, linkage to HIV treatment and care services, and staging and antiretroviral therapy (ART) initiation, and advancement as it pertains to education and job training, as measured by enrollment in training certifications, self-reported motivation and perception regarding learning, and self-reported knowledge regarding strategies to improve educational outcomes.

### Staff training

The intervention facilitators, including the EPWPs, are trained by the Principal Investigator or the South African Project Director on the intervention workshops. All project staff involved are trained on the research methods, data collection, and issues surrounding confidentiality. Further, all field staff receive a field manual and training on safety and security procedures, protection of human subjects, and how to conduct active and passive referrals.

### Data management and quality assurance

To protect participants’ confidentiality, several procedures are implemented at both the field site in South Africa and at RTI headquarters in the United States. All study participants receive a computer-generated participant ID that is used on all study-related data in paper and electronic form. The participant ID contains a check digit and all data collection software verifies that only valid participant IDs are entered. Project files and databases associated with this study are only available to research personnel through the authorisation of the project’s Principal Investigator. All locator information is stored in double-locked cabinets in the field site location.

CAPI and ACASI are used for the personal data collection, allowing for checking errors in real time. ACASI provides the privacy for collecting more sensitive data and the advantage of having the interviewer offer to assist with question comprehension. Data management is conducted by RTI’s Research Computing Division. Data are transmitted daily to RTI and processed nightly, with reports sent to project team members. An edit report identifies data issues needing attention, a tracker report displays key information from data records for each participant, and a production report prints needed statistics to evaluate study progress.

Biological data are collected for pregnancy, AOD use, and HIV through urinalysis, breath scans, and blood specimen collection. There is no storage of biological specimens because they are immediately disposed of as medical hazard waste following testing.

### Adverse events and severe adverse events

If participants experience AEs or SAEs in direct relation to any aspects of the study, the South African Project Director will complete an Incident Report immediately and contact the South African Co-Investigator and the Principal Investigator for additional guidance, as needed. The field staff will make appropriate referrals to medical, counselling and/or other health services. SAE and Incident Reports will be reviewed by the Principal Investigator as soon as they occur. SAEs, such as death or hospitalisation, will be reported within 24 h of their occurrence by the Principal Investigator to the National Institute on Drug Abuse Project Officer, RTI’s Office of Research Protection, the SAMRC Ethics Committee, and the DSMB described earlier.

### Sample size and power

#### Power considerations for Aim 2

This study proposes several primary outcomes for Aim 2, and the power analysis were conducted to ensure that the study was adequately powered to detect intervention effects on these key outcomes, such as proportion of condom use (see earlier description of primary and secondary outcomes). The assumptions for this analysis are based on past studies of the WHC in South Africa and the Young Women’s CoOp pilot efficacy study conducted in the United States. In past studies, the proportion of condom use has ranged from 0.3 to 0.6. For all power analysis, we assumed a two-sided test with α = 0.05, a power of 0.8, and an intracluster correlation coefficient (ICC) estimate of 0.01. Based on these parameters, with a sample size of 225 per intervention arm at 12-month follow-up (an expected 90% follow-up) and 24 clusters, the minimal detectable difference is 0.13 for the outcome of condom use at the highest proportion of 0.6. For outcomes that may be less common overall, such as biologically confirmed HIV, we will be able to detect similar differences, ranging from 0.10 to 0.13.

### Analysis

To address Aim 1, we will analyse data collected by both peer role models and project staff when conducting daily activities in the field. Simple descriptive data analysis will be used to answer this aim, such as counts of marketing materials distributed, counts of eligible young women introduced to project staff by peer role models and screened eligible. However, our primary analysis will test the hypothesis that the YWHC will reduce the primary outcome (sexual risk behaviour) as well as AOD use and victimisation relative to standard HCT (Aim 2: Hypothesis 1).

To test the efficacy of the intervention on each of these outcomes, we will use a standard intention-to-treat approach by including all participants as assigned to their study arm, regardless of their intervention exposure. The study design, which is longitudinal and clustered by community, creates an inherent multilevel structure, thereby introducing nonindependence. Consequently, our analytic strategy will be based on generalised linear mixed models, as use of hierarchical or multilevel mixed models allows for clustering due to community-level randomisation and repeated measures within individuals over time (at 6- and 12-month follow-up). Primary models will include a covariate for study arm, adjust for the baseline level of the outcome (e.g., heavy alcohol use), and account for community- and individual-level clustering. We will evaluate intervention effects individually at the two time points, initially by calculating proportions of the primary outcomes in the two randomised groups, with 95% confidence intervals. For multivariable modelling of intervention effects, we will use logit, Poisson, and linear regression approaches, as appropriate.

We will also test Hypothesis 2, which posits that the young women’s comprehensive HCT programme will improve the secondary outcome—access to HIV, STI, antenatal and reproductive health services—relative to standard HCT, using a similar data analysis approach. We will use a standard intention-to-treat analysis by including all participants as assigned to their study arm, regardless of their intervention exposure. The primary outcome of improved access will be operationalised in two ways: (1) a dichotomous measure of whether services were accessed (any and by type of service); and (2) a count of the number of times services were used during the 12-month follow-up period.

As described above, the initial test of the hypothesis that adolescent women in the YWHC intervention arm—as compared with adolescent women in the standard HCT arm—will be more likely to seek reproductive health services over 12 months, will be conducted with a model that includes a main (fixed) effect for intervention arm, a baseline measure of previous use of reproductive health services, and a random effect for community. We will conduct an exploratory analysis that includes potential effect modifiers and mediators—such as family connections, neighbourhood gang activity, and housing instability—to assess which interpersonal and structural factors might influence the YWHC intervention’s effects on access to HIV and other services.

## Discussion

Few evidence-based programmes exist that target adolescent women who are out of school. This article describes the study protocol for a project to reduce AOD use, victimisation, and sexual risk behaviour and to increase uptake of HCT services for out-of-school adolescent women who use AODs. South Africa’s historical ties to AOD use, poverty, violence, and gender inequality are prominent in many of the communities where these adolescent women live. This project provides an opportunity to reach vulnerable adolescent women who have dropped out of school and empower them through a gender-focused approach.

The use of peers to assist with recruitment and cofacilitate the intervention is novel and may increase the intervention’s appeal to adolescent women and the chances of them participating in this study. The study findings will increase knowledge of the efficacy of a comprehensive HCT, gender-focused programme in reducing risk behaviours and increasing uptake services for out-of-school adolescent women who use AODs. Overall, the study could lead to a larger implementation of the YWHC for vulnerable adolescent women, a key population often neglected in health services.

### Trial status

Enrolling.

## Abbreviations

ACASI: Audio computer-assisted self-interviewing
AEs: Adverse events
AOD: Alcohol and other drugs
ART: Antiretroviral therapy
ARV: Antiretroviral
CAPI: Computer-assisted personal-interviewing
CITI: Collaborative Institutional Training Initiative
DSMB: Data and Safety Monitoring Board
DSMP: Data and Safety Monitoring Plan
EPWP: Expanded Public Works Programme
GBV: Gender-based violence
HCT: HIV counselling and testing
ICC: Intracluster correlation coefficient
N3: National Technical Certificate
RRBA: Revised Risk Behavior Assessment
SAEs: Severe adverse events
SAMRC: South African Medical Research Council
STIs: Sexually Transmitted Infections
WHC: Women’s Health CoOp
YRBS: Youth Risk Behaviour Survey
YWC: Young Women’s CoOp
YWHC: Young Women’s Health CoOp
